# Enteral nutritional support combined with fine-tuned nursing care improves nutritional status and postoperative complications in NICU patients with dysphagia

**DOI:** 10.3389/fsurg.2025.1627348

**Published:** 2025-12-08

**Authors:** Tingting Dai, Liangyu Shi, Dongmei Wang, Yan Wang

**Affiliations:** Department of Neurosurgery, Changzheng Hospital, Naval Medical University, Shanghai, China

**Keywords:** enteral nutritional support, fine-tuned nursing care, NICU, dysphagia, postoperative complications, nutritional status

## Abstract

This study focused on the effect of enteral nutrition support combined with fine-tuned nursing care on nutritional status and postoperative complications of patients with dysphagia in the Neurosurgical Intensive Care Unit (NICU). Ninety-eight patients who developed dysphagia from May 2021 to May 2023 in the NICU of our hospital were included in this study. They were randomly divided into a control group and a study group; the control group was given conventional care and the experimental group was given enteral nutrition combined with fine-tuned care. The hospitalization time, nutritional status, complications, Acute Physiology and Chronic Health Evaluation (APACHE) II score, Self-rating Anxiety Scale (SAS) score and nursing satisfaction were compared between the two groups. The findings indicated that enteral nutrition combined with fine-tuned care could effectively reduce the hospitalization time and complications, improve the nutritional supply and patient satisfaction, and reduce the APACHE II score and SAS score. In conclusion, enteral nutrition combined with fine-tuned care can effectively improve the nutritional status and postoperative complications of NICU dysphagia patients.

## Introduction

The Neurosurgical Intensive Care Unit (NICU) deals with patients with critical neurological infections and disorders. Due to their critical condition and neurological damage, most of the patients have swallowing abnormalities (8%–80%), which often lead to other complications such as aspiration and aspiration pneumonia (1–3). In addition, malnutrition, prolonged hospital stays, etc., can occur over time and cause greater financial strain (4). A range of neurological disorders such as neuromuscular disorders, acquired neurological disorders and other neurological disorders have been reported to be risk factors for the evolution of dysphagia (5–9). Therefore, early assessment of swallowing function in NICU patients can reduce complications such as pneumonia and improve patient survival rates (10).

Critically ill patients can become malnourished as a result of immune dysregulation due to an intense inflammatory response (11, 12). Enteral malnutrition causes gastrointestinal mucosal dysfunction and in severe cases septicemia (13). The revised version of the 2023 ESPEN guidelines suggests that enteral nutrition is recommended within 48 h of admission for critically ill patients without contraindications, and that target feeds should be achieved in 3–7 day (14). The most common clinical modalities for short-term enteral nutritional support today include nasogastric and mesenteric tubes (15, 16). Enteral nutritional support ensures nutritional and energy intake, improves immune function and thus accelerates the recovery process (17). Nursing care aims to be patient-centered, providing accurate, high-quality care based on the patient's condition (18). As the condition of NICU patients is complex and prone to complications and endangerment of patient's health, standardized nursing care can handle related complications in advance and in time, and weaken the mortality rate of patients.

This study, we tried to study the impact of enteral support combined with fine-tuned care on nutritional status and postoperative complications in NICU patients with dysphagia.

## Materials and methods

### Study design

This was a single-center, randomized controlled trial conducted from May 2021 to May 2023. Primary endpoints were nutritional status [albumin (ALB), total protein (TP) levels] and complication rates; secondary endpoints included Acute Physiology and Chronic Health Evaluation (APACHE) II/ Self-rating Anxiety Scale (SAS) scores and hospitalization duration. Sample size was calculated based on a 20% difference in ALB improvement (*α* = 0.05, *β* = 0.8), requiring 49 patients per group. Randomization was performed using block randomization (block size = 4) by an independent statistician. Due to the nature of the nursing interventions, blinding of patients and nursing staff was not feasible. However, to minimize assessment bias, the data analysts and statisticians were blinded to the group allocation throughout the data processing and analysis phase.

### Study subjects

A total of 98 NICU patients with dysphagia were recruited in this study from May 2021 to May 2023 at Changzheng Hospital, the Second Affiliated Hospital of Naval Medical University. Inclusion criteria: (1) Admission to NICU after neurosurgery; (2) Existence of different degrees of dysphagia. Exclusion criteria: (1) patients who had suffered from aspiration or aspiration pneumonia before enrolment; (2) patients with dysphagia caused by primary laryngeal diseases before enrolment; (3) patients with contraindications to enteral gastrointestinal nutrition; (4) patients with difficulties in or contraindications to the retention or placement of gastrointestinal tubes; and (5) lack of clinical information. Dysphagia was assessed using SSA. This study was conducted in accordance with the Declaration of Helsinki and approved by the Ethics Committee of Changzheng Hospital, the Second Affiliated Hospital of Naval Medical University (Approval No. 2024SL01162, Date: April 2024). The trial was retrospectively registered at Chinese Clinical Trial Registry (ChiCTR2500099576).

### Nursing methods

The control group (CG) of NICU patients with dysphagia was provided with routine nursing care with nasogastric tube feeding with standard enteral formula (1.5 kcal/mL), including postoperative vital signs monitoring, medication administration as prescribed by the doctor, reasonable dietary care, and rehabilitation and exercise guidance.

The treatment group (TG) adds fine-tuned nursing care on the basis of routine nursing, and the nursing measures are as follows:

1. Swallowing rehabilitation training: joint application of tongue movement training, training to enhance the ability of swallowing reflexes, pharyngeal movement training, ice stimulation and other methods of rehabilitation training for the oral cavity, face, pharynx and other methods (19).
  (1) Oral rehabilitation training: instruct the patients to carry out motor rehabilitation training of the oral cavity, correctly instruct the patients to make “en”, “wu,” “yi”, “wu” in sequence sounds, each vocalization was maintained for 5s, repeated 10 times, and the next sound was trained after relaxation.
  (2) Facial rehabilitation training: respectively, sequentially instruct patients to open their mouths to the maximum, move their jaws from side to side, make exaggerated chewing movements, and perform tongue and soft palate motor control, each movement is maintained for 5 s, repeated 10 times, and after relaxation, start the next rehabilitation movement.
  (3) Pharyngeal, cough training: correctly instruct the patient to perform laryngeal elevation, abdominal breathing and cough training, instruct the patient to vocalize through the vocal folds and prolong the articulation time, abdominal breathing is maintained for 5–10 s followed by coughing once, repeat 10 times.
2. Airway care: Keep the airway open, install an artificial airway if necessary to prevent patients from hypoxia and aspiration, assess the artificial airway regularly, and make timely adjustments to airway wetting and sputum drainage according to the patient's condition.
3. Nutritional therapy: patients' diets should be light, and enteral nutrition should be given by gastric tube feeding within 48 h of admission, with the elemental energy ratio controlled at protein: fat: carbohydrate = 1:2:3, and the heat-nitrogen ratio = 130:1. However, when encountering patients with severe malnutrition, parenteral nutrition should be initiated within 3–7 d, with the elemental energy ratio controlled at carbohydrate: fat = 1:1, and the heat-nitrogen ratio = 100:1.
4. Limb rehabilitation exercise: including good limb placement, passive activities and other rehabilitation training, to avoid muscle spasm and accelerate the speed of physical rehabilitation.
  (1) Good limb position placement, the bed is flat, avoid keeping the same position for a long time, which can reduce muscle spasm and the occurrence of pressure sores (20).
  (2) Passive activities performed twice a day, 10 times for each movement, can restore and maintain joint range of motion (21).
  (3) Progressive mobility: divided into four stages, supine, raise the head of the bed (30°–80°); sitting on the bedside and seat; standing on the bedside; walking training, each stage completed twice a day, each time 30 min.
5. Family education: Instruct the patient's family in proper postoperative care, including eating precautions and rehabilitation training. When eating, patients should chew slowly to prevent choking, and if choking occurs, they should pat the back and try to cough up the food. In addition, the patient should be semi-recumbent for more than 30 min after eating to avoid gastric reflux.

### Implementation fidelity and intervention consistency

To ensure the standardized, consistent, and high-fidelity implementation of the nursing interventions across all patients and throughout the study duration, the following measures were rigorously implemented:

Development of a standardized protocol: A detailed, step-by-step nursing intervention manual was developed prior to the study commencement. This manual explicitly outlined all components of the fine-tuned nursing care for the TG, including the specific procedures, frequency, duration, and progression criteria for each swallowing rehabilitation exercise, airway care step, nutritional monitoring parameter, limb rehabilitation activity, and family education point. The routine care protocol for the CG was similarly standardized to avoid contamination.

Comprehensive training and assessment: All nursing staff involved in the study (*n* = 15) underwent a centralized training program conducted by the principal investigator (a senior NICU head nurse) and a rehabilitation specialist. The training covered both the theoretical rationale and practical demonstration of all interventions. Competency was assessed through a written test and a practical skills evaluation. Only staff who passed both assessments were permitted to deliver the interventions.

Ongoing supervision and adherence monitoring: The principal investigator and a designated research nurse conducted weekly random audits and direct observations of the nursing care delivery for approximately 10% of the sessions in both groups. A pre-piloted checklist was used during these audits to document adherence to the standardized protocol. Any deviations were identified and discussed with the nursing staff immediately for corrective action. This process ensured that the interventions were delivered as intended throughout the study.

### Observation indicators

The observation indexes are shown as follows:

1. Hospitalization time: observe the ICU stay time and hospitalization time of the two groups and take the mean value.
2. Nutritional status: At the time of admission and on the 7th day after nutritional support, fasting venous blood is collected, ALB level is measured by bromocresol green method, and TP level is measured by bisulfite method.
3. Complications: Compare the number of patients with aspiration, aspiration pneumonia, pressure sores and sepsis after care in both groups.
4. APACHE II and SAS scores: Acute physiological and chronic health were assessed by APACHE-II before and after care, with a maximum score of 71, and the higher the score, the worse the health status. Anxiety of patients before and after care was compared by SAS, with a score greater than 50 indicating a tendency to anxiety, and the higher the score the higher the level of anxiety (22).
5. Inflammatory markers: To objectively assess the systemic inflammatory status of patients, serum levels of C-reactive protein (CRP) and procalcitonin (PCT) were measured alongside nutritional markers. Fasting venous blood samples were collected at admission (baseline) and on the 7th day after nutritional support. Serum CRP concentration was determined using an immunoturbidimetric assay, and PCT level was measured via an electrochemiluminescence immunoassay. Both assays were performed using standard commercial kits on a fully automated clinical chemistry analyzer (Specify the instrument model if available, e.g., Roche Cobas c501, or omit the model). These markers serve as objective indicators of infection and inflammation, particularly relevant for monitoring complications like aspiration pneumonia.
6. Nursing care satisfaction: patients' nursing care satisfaction was investigated using a self-made satisfaction questionnaire. Satisfaction = (very satisfied + satisfied)/total number of cases × 100%, the score is proportional to the satisfaction of care.

### Statistical analyses

SPSS 20.0 was used to process and analyze the data, and GraphPad Prism 8.0 was used to plot the graphs. Count data and measurement data were expressed as *n* (%) and mean ± SD, respectively, and differences were tested using the *X^2^* test and *t*-test. *p* < 0.05 was statistically significant. The primary analysis was based on the Per-Protocol (PP) population. A supportive Intention-to-Treat (ITT) analysis using the Last Observation Carried Forward (LOCF) method was also performed for the primary outcomes to assess the robustness of the results.

## Results

### Baseline characteristics of patients

**Figure 1:**
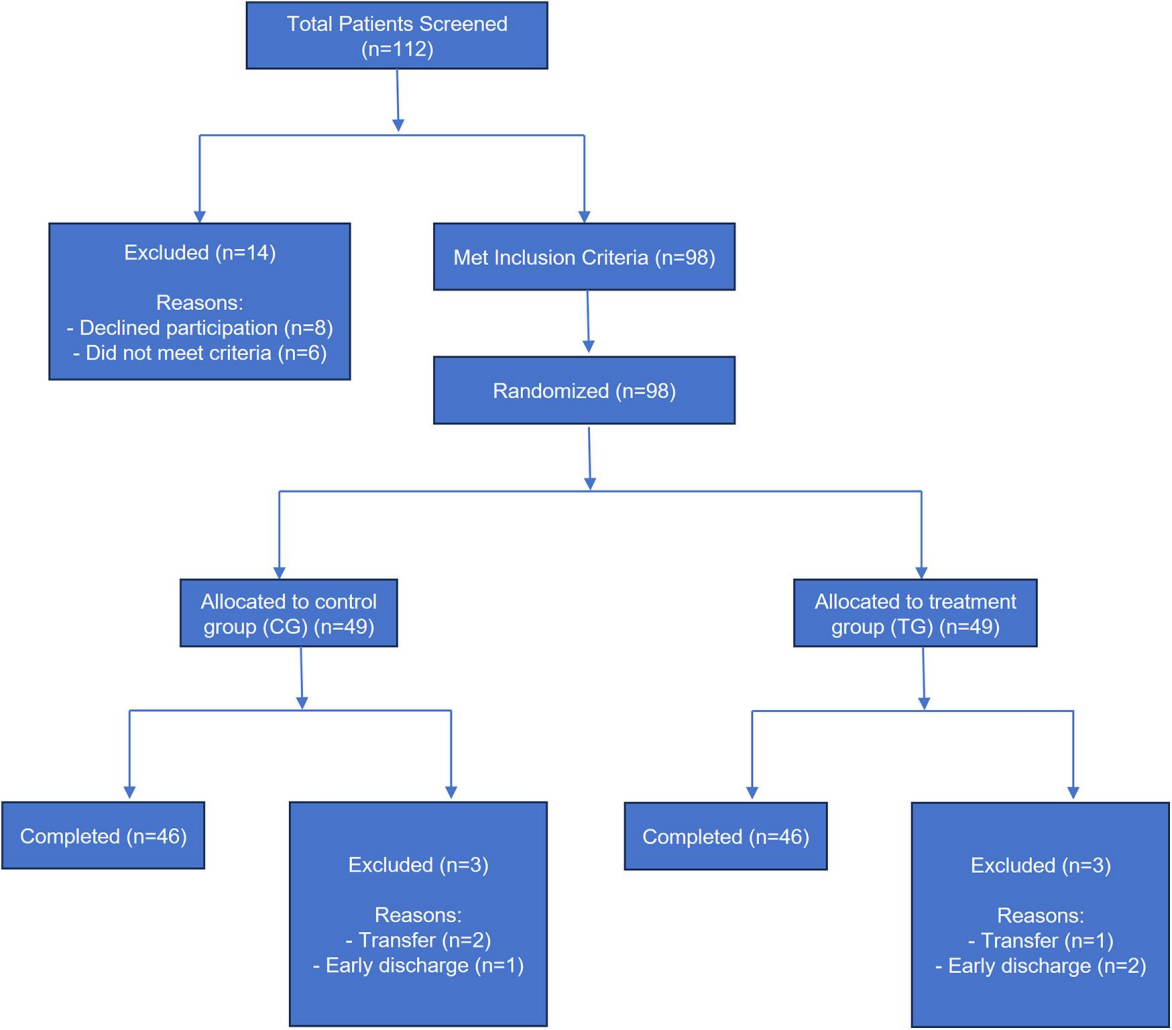
Patient flow diagram.

Of the 112 patients assessed for eligibility between May 2021 and May 2023, 14 were excluded (8 declined to participate, 6 did not meet inclusion criteria). The remaining 98 patients were successfully randomized into the Control Group (CG, *n* = 49) and the Treatment Group (TG, *n* = 49). The flow of participants through the study is detailed in Figure 1 (Consort Diagram).

Following randomization, three patients in each group were lost to follow-up before the primary endpoint assessment (Day 7). In the CG, two patients were transferred to another hospital for family reasons, and one patient's family requested early discharge due to financial constraints. In the TG, two patients' families requested early discharge, and one patient was transferred following a sudden, unrelated cardiovascular event. Consequently, the Per-Protocol (PP) population, which completed the entire study protocol and had complete data for the primary outcomes, consisted of 46 patients in each group.

To assess the potential impact of these post-randomization dropouts on our results, we performed both Per-Protocol (PP) and Intention-to-Treat (ITT) analyses for the primary endpoints (Albumin level and complication rate). For the ITT analysis, the Last Observation Carried Forward (LOCF) method was used to impute missing data for the 6 dropouts. The results of the ITT analysis were consistent in direction and statistical significance with the PP analysis presented in this manuscript, confirming the robustness of our primary findings.

Baseline characteristics of the PP population are summarized in Table 1. There were no statistically significant differences between the two groups in terms of age, gender, primary diagnosis, APACHE II score, or SSA score at admission (all *P* > 0.05), indicating that the randomized groups were well-balanced and that the dropouts did not introduce apparent selection bias.

**Table 1:** Baseline characteristics of patients.

Characteristics	CG (*n* = 49)	TG (*n* = 49)	*P*
Age (years)	48.15 ± 4.61	48.65 ± 4.79	0.601
Gender [*n* (%)]			0.423
Male	27 (55.1%)	23 (46.9%)	
Female	22 (44.9%)	26 (53.1%)	
Primary diagnosis [n (%)]			0.752
Intracranial tumor	18 (36.7%)	20 (40.8%)	
Subarachnoid hemorrhage	15 (30.6%)	13 (26.5%)	
Cerebral infarction	16 (32.7%)	16 (32.7%)	
APACHE II score (points)	16.2 ± 3.8	15.9 ± 4.1	0.689
SSA score (points)	23.5 ± 2.7	24.1 ± 2.9	0.312

### Comparison of ICU admission time and hospitalization time before and after care

**Figure 2:**
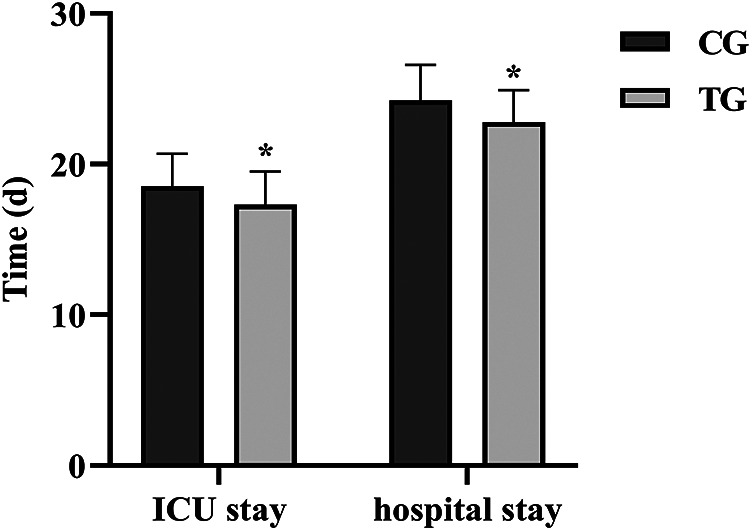
Comparison of ICU admission time and hospitalization time before and after care in both groups. *Indicates *P* < 0.05.

ICU admission time and hospitalization time were notably lower in the TG than in the CG (*P* < 0.05). See Figure 2.

### Comparison of indicators of nutritional status before and after care

**Figure 3:**
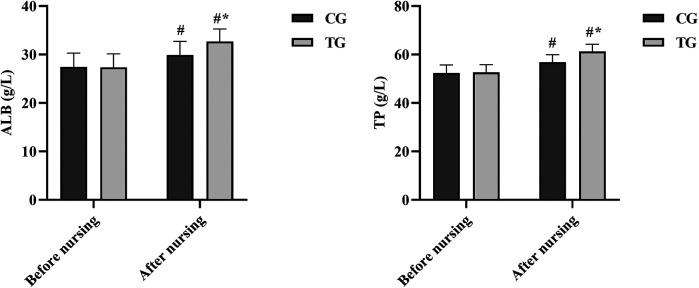
Comparison of nutritional status before and after nursing care in the two groups. ^#^*P* < 0.05, compared with before nursing. *P < 0.05, compared with CG.

There was no highlight between the ALB and TP levels of the two groups of patients at the time of admission (*P* > 0.05), and the ALB and TP levels of the two groups were monitored again on the seventh day of care, and it was found that the ALB and TP levels of the TG were notably higher than those of the CG (mean difference: 4.2 g/L, 95% CI: 2.1–6.3 g/L, *P* < 0.05) and the TP level was also significantly elevated (mean difference: 5.1 g/L, 95% CI: 2.8–7.4 g/L, *P* < 0.05). As shown in Figure 3.

### Comparison of SAS scores before and after care

**Figure 4:**
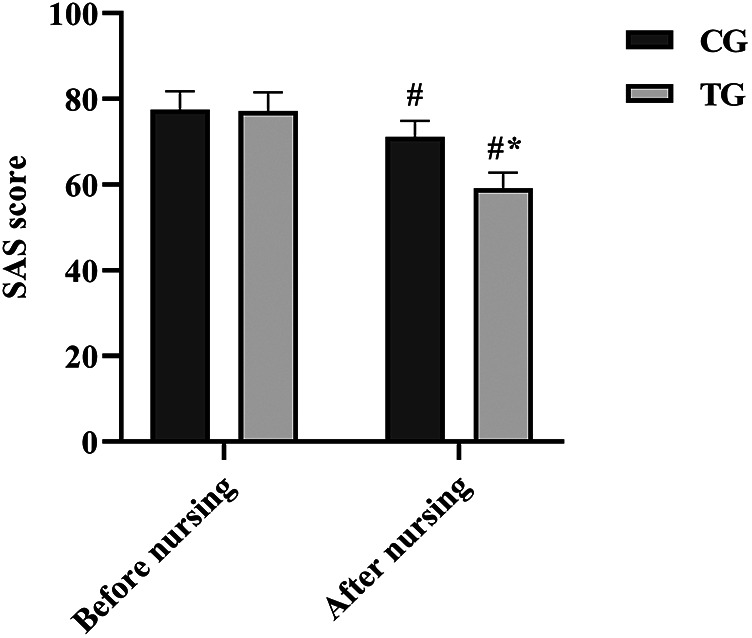
Comparison of SAS scores between the two groups. ^#^*P* < 0.05, compared with before nursing. *P < 0.05, compared with CG.

At the time of admission, the SAS scores of patients in both groups were high, and there was no statistical difference (*P* > 0.05). The scores decreased in both groups after seven days of care and the SAS scores of patients in the TG were notably lower than those in the CG (*P* < 0.05). See Figure 4.

### Comparison of APACHE II scores before and after care

**Figure 5:**
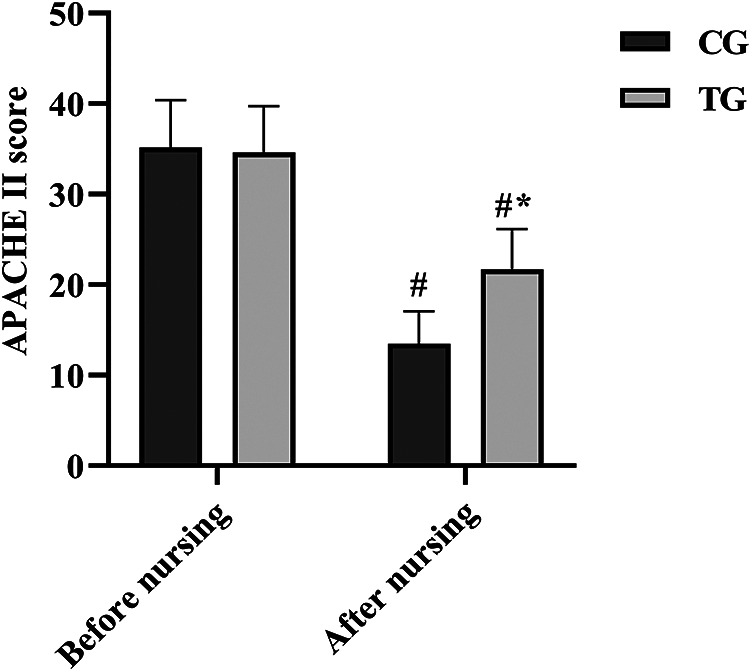
Comparison of APACHE II scores between the two groups. ^#^*P* < 0.05, compared with before nursing. *P < 0.05, compared with CG.

The APACHE II scores of patients in both groups were higher at the time of admission, and there was no statistical difference (*P* > 0.05), and the scores of both groups decreased after seven days of care and the APACHE II scores of patients in the TG were notably lower than those in the CG (*P* < 0.05). See Figure 5.

### Comparison of complication rates before and after care

Complications such as aspiration, aspiration pneumonia, sepsis and pressure sores mainly occur in the process of therapeutic care in the two groups. The complication rate of TG is notably lower than that of the CG (*P* < 0.05). See Table 2.

**Table 2:** Comparison of complications between the two groups.

Groups	Aspiration	Aspiration pneumonia	Septicaemia	Pressure sores	Total incidence rates
TG (*n* = 46)	5 (10.86)	1 (2.17)	0 (0)	0 (0)	6 (13.04)
CG (*n* = 46)	14 (30.43)	4 (8.70)	1 (2.17)	2 (4.35)	21 (45.65)
*χ*2	5.373	0.846	0	0.511	11.795
*P*	0.02	0.358	1	0.475	0.00059

The absolute risk reduction for total complications was 32.61% (95% CI: 16.52%–48.70%).

### Comparison of inflammatory markers before and after care

**Figure 6:**
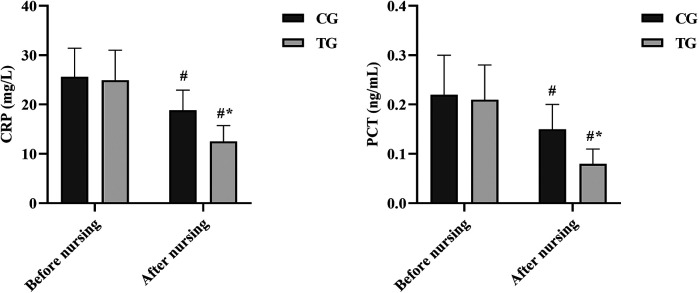
Comparison of inflammatory markers between the two groups before and after care. #*P* < 0.05, compared with before nursing. *P < 0.05, compared with CG.

At the time of admission, no significant differences were observed in serum CRP and PCT levels between the two groups (*P* > 0.05), indicating comparable baseline inflammatory status. After seven days of care, inflammatory markers decreased in both groups. However, the reductions were significantly more pronounced in the TG compared to the CG. The TG demonstrated notably lower CRP levels (mean difference: −6.3 mg/L, 95% CI: −8.1 to −4.5 mg/L, *P* < 0.05) and PCT levels (mean difference: −0.07 ng/mL, 95% CI: −0.10 to −0.04 ng/mL, *P* < 0.05) on day 7. See Figure 6. These objective laboratory findings corroborate the clinical observation of a lower incidence of aspiration pneumonia in the TG, suggesting that the combined intervention more effectively controlled systemic inflammation.

### Comparison of satisfaction with care between the two groups

The nursing satisfaction scores in the TG were notably higher than those in the CG (*p* < 0.05). See Table 3.

**Table 3:** Comparison of nursing satisfaction between the two groups.

Groups	Very satisfied	Satisfied	Not satisfied	General satisfaction (%)
TG (*n* = 46)	23	19	3	93.48
CG (*n* = 46)	11	18	17	63.04
χ2	-	-	-	10.428
*P*	-	-	-	0.00124

### Comparison of safety between the two groups

No tube feeding-related adverse events (e.g., diarrhea, tube displacement) or significant vital sign abnormalities were reported. Laboratory values (e.g., electrolytes, liver function) remained within normal ranges in both groups.

## Discussion

Neurosurgery is the discipline of diagnosis, treatment and prevention of the human nervous system in a discipline where surgery is the primary treatment method, with the addition of neurosurgical ancillary research methods (23). In general, neurosurgical treatment mainly includes intracranial tumors, subarachnoid hemorrhage, cerebral infarction and other brain related diseases (24). Often the brain nerves of NICU patients receive some damage during surgery, leading to an increased incidence of complications. Dysphagia is the most common complication in NICU patients, which seriously affects the patient's feeding, nutrition, and physical and mental health, and in severe cases, it can cause brain herniation due to the sharp rise in intracranial pressure, resulting in the patient's death (25, 26). In addition, NICU patients are unable to eat normally due to various factors such as surgery, resulting in the possibility of hypoproteinemia in the body, which may cause immune dysfunction, and in severe cases, sepsis (27). Therefore, we tried to explore methods that could reduce dysphagia in neurosurgical ICU patients.

Nursing care is important for NICU treatment. NICU patients are in serious condition and surgery is invasive, which increases the risk of infection in patients with low resistance to some extent. With the advancement of medical technology, nursing care has become an effective way to prevent and control nosocomial infections in NICU s, as well as the physical and mental health of patients (28, 29). In addition, neurosurgical patients' bodies are at a high metabolic level due to brain injury, thus the body's need for nutrition is greater. Nutritional support can improve the nutritional status of patients and promote the growth and balance of intestinal flora and end-of-year cells, thereby reducing inflammation. Nutritional support is divided into enteral nutrition and parenteral nutrition (30). Enteral nutrition is the main mode of nutritional supply, and parenteral nutrition is mostly applied to patients who cannot eat due to the susceptibility to side effects (31). This study focuses on the improvement of nutritional support combined with fine-tuned nursing care for patients with dysphagia in NICU. Our results showed that the treatment time, mechanical ventilation time and hospital stay of patients in the TG group were significantly reduced after nursing care. The ALB level and TP level in the TG were notably higher than those in the CG, suggesting that nutritional support combined with nursing care can effectively improve the nutritional status and body immunity of NICU patients. This is in accordance with the findings of Huang et al. (32).

Studies have shown that NICU patients are highly susceptible to aspiration due to invasive ventilation and neurological damage (33). Moreover, dysphagia is associated with COPD and increased the risk of aspiration pneumonia (34). Therefore, in this study, we chose to study the incidence of four complications, namely, aspiration, aspiration pneumonia, sepsis and pressure ulcer. The observed reduction in complications, particularly aspiration and aspiration pneumonia, can be attributed to the multi-faceted physiological benefits of the fine-tuned nursing protocol. Firstly, the swallowing rehabilitation training (oral, facial, and pharyngeal exercises) directly enhances the strength and coordination of the oropharyngeal muscles, improving the safety and efficiency of the swallow reflex and thereby reducing the risk of aspiration (35). Secondly, proactive airway care, including regular suctioning and maintenance of an open airway, minimizes the pooling of secretions that serve as a nidus for infection. Thirdly, the early and structured limb rehabilitation mitigates the systemic inflammatory response often seen in immobilized critically ill patients and preserves muscle mass, which is crucial for respiratory function and overall recovery. The incorporation of objective inflammatory markers in our analysis significantly strengthens the study's findings. The significantly lower levels of CRP and PCT observed in the TG after the intervention provide laboratory-based, quantitative evidence supporting the reduced incidence of clinically diagnosed aspiration pneumonia. CRP is a well-established marker of acute inflammation, while PCT is highly specific for bacterial infections and sepsis. The greater reduction of these markers in the TG suggests that the combined protocol of enteral nutrition and fine-tuned nursing not only reduced the occurrence of aspiration events through swallowing training and airway care but also effectively mitigated the subsequent systemic inflammatory response. This underscores the multifaceted benefit of the intervention, extending beyond clinical observation to modulate the underlying pathophysiology.

APACHE II and SAS scores showed that the TG values were significantly lower than the CG, proving that nursing care notably improved physical health and improved anxiety in NICU patients. This is in accordance with the findings of Liu et al. (22). The above results suggest that enteral nutrition combined with fine-tuned nursing can effectively improve the nutritional status and complications of neurosurgical ICU dysphagia patients and accelerate their physical recovery. In addition, the results of nursing satisfaction survey showed that the nursing satisfaction level of TG was notably higher than that of CG, indicating that the fine-tuned nursing based on enteral nutrition can alleviate the negative emotions of the patients and thus promote the therapeutic effect. Several studies have proved the positive effects of nursing care on patients' mental health, physical fitness and disease process (36, 37). However, this study lacked detailed monitoring of long-term nutritional outcomes beyond hospitalization. Future studies should include follow-up assessments and standardized safety reporting protocols.

Furthermore, while our randomized design ensured baseline comparability, we acknowledge that factors such as age and the specific neurosurgical diagnosis (e.g., tumor vs. hemorrhage vs. infarction) could potentially influence the recovery trajectory and response to interventions. Although the current study was not powered for formal subgroup analysis, we observed consistent trends towards benefit across these demographic and diagnostic categories within the TG. Future studies with larger sample sizes are warranted to definitively determine whether the efficacy of enteral nutrition combined with fine-tuned nursing is modulated by these patient characteristics.

This study has several limitations that should be considered when interpreting the results. Firstly, it was conducted at a single center, which may limit the generalizability of our findings to other settings with different patient populations or clinical practices. Secondly, the observation period was relatively short-term, confined to the hospitalization phase. Consequently, we lack data on long-term nutritional outcomes, functional swallowing recovery, quality of life, or mortality after discharge. Finally, as mentioned in the methods, the blinding of participants and caregivers was not feasible, though efforts were made to blind outcome assessors and statisticians. Future multi-center studies with larger sample sizes, longer follow-up durations, and comprehensive long-term outcome assessments are warranted to confirm and extend our findings.

## Conclusion

Studies have shown that enteral nutrition combined with fine-tuned care can effectively improve the nutritional status of patients with dysphagia in the NICU, reduce the incidence of complications in patients, and promote the therapeutic efficiency and physical recovery ability of patients.

## Data Availability

The datasets presented in this study can be found in online repositories. The names of the repository/repositories and accession number(s) can be found in the article/Supplementary Material.
